# Subsolid Nodule Harbouring Extranodal Marginal Zone Lymphoma of Mucosa-Associated Lymphoid Tissue

**DOI:** 10.5334/jbsr.2915

**Published:** 2022-12-08

**Authors:** Stijn Marcelis, Amélie Dendooven, Annemiek Snoeckx

**Affiliations:** 1 UZA, BE; 2 Ghent University Hospital and University of Ghent, BE; 3 Antwerp University Hospital and University of Antwerp, BE

**Keywords:** Subsolid, nodule, lymphoma, MALT, CT, PET

## Abstract

With the widespread use of computed tomography (CT), subsolid nodules are more frequently encountered in daily practice. We present the case of a 74-year-old man with a large persistent well-defined subsolid nodule on CT. Although the lesion had a predominant ground-glass appearance on CT, ^18^F-fluorodeoxyglucose (FDG) positron emission tomography (PET) showed moderate FDG uptake. Lobectomy was performed and histopathologic examination showed an extranodal marginal zone lymphoma of mucosa-associated lymphoid tissue.

**Teaching Point:** When large persistent subsolid nodule with a predominant ground-glass aspect shows moderate uptake on ^18^F-FDG-PET, other possible diagnoses than adenocarcinoma should be kept in mind, including primary pulmonary lymphoma.

## Introduction

Primary pulmonary lymphoma is a rare, malignant monoclonal lymphoid proliferation within the lung parenchyma. On imaging it can have different imaging appearances, including single or multiple lesions, nodules and masses, and/or areas of consolidation or ground glass. Whereas subsolid pulmonary nodules most often correlate on histopathology to adenocarcinoma with lepidic growth, in rare cases these lesions may represent primary pulmonary lymphoma.

## Case History

**Figure 1:**
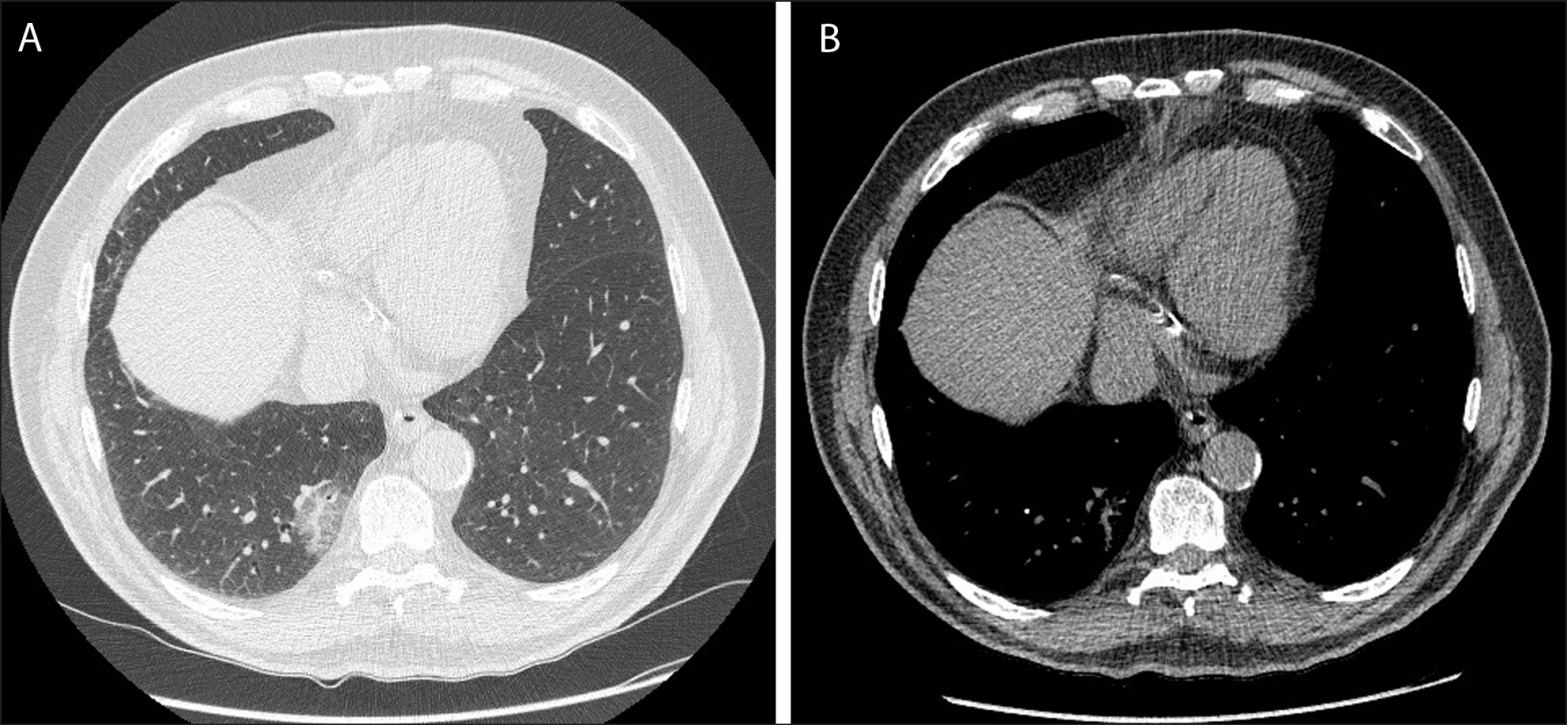
Non-contrast-enhanced CT image of the chest. Axial image in lung window setting **(A)** shows a well-delineated subsolid nodule in the paravertebral region of the right lower lobe. The lesion has a predominant ground glass appearance. A more prominent vessel is running through the lesion, but in mediastinal setting **(B)** no clear solid components can be delineated.

**Figure 2:**
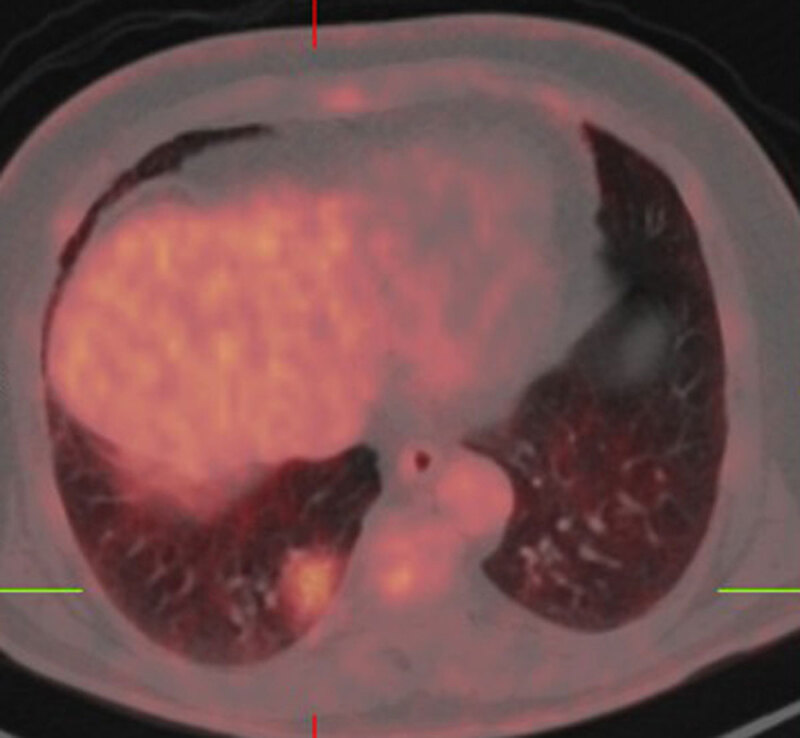
Fused ^18^FDG-PET/CT image in axial plane shows moderate FDG uptake in the subsolid nodule in the right lower lobe.

**Figure 3:**
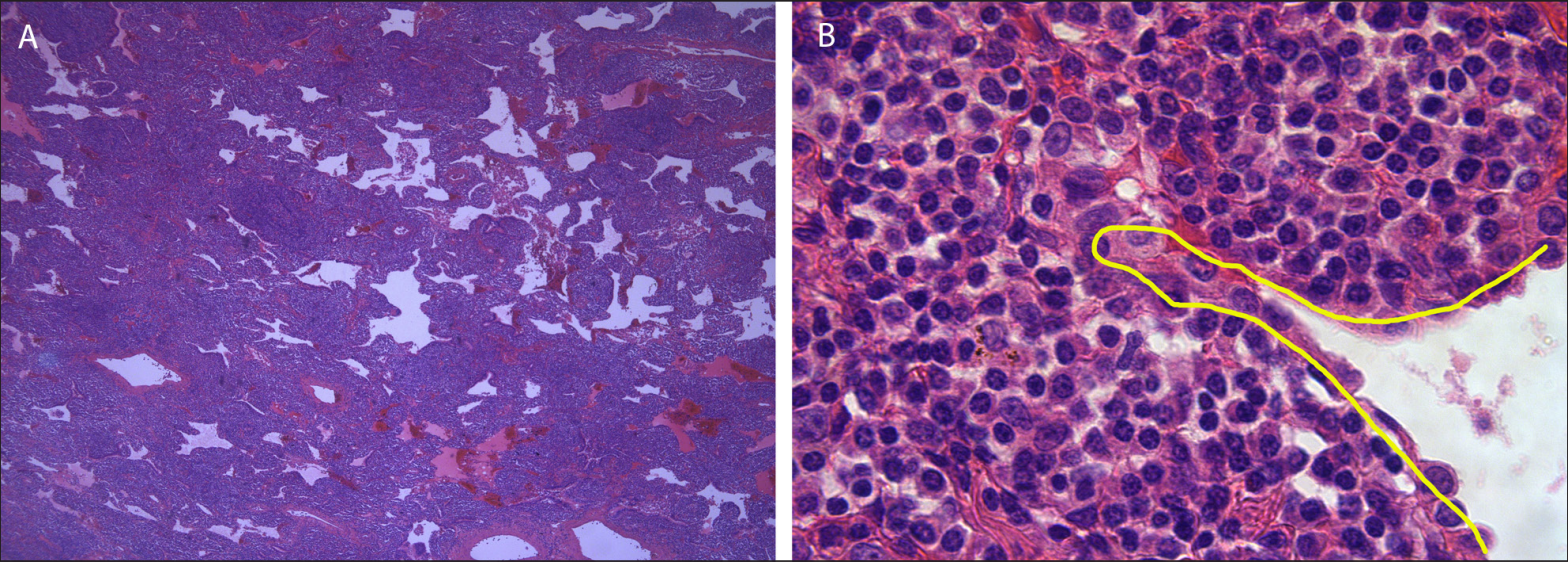
Photomicrographs of the resected specimen. Overview **(A)** shows the lesion consisting of diffusely strongly widened septa infiltrated by the tumoral lymphoid cells. Importantly, the tissue architecture is densened by the tumoral infiltrate. Close-up image **(B)** shows the tumor in the alveolar septa. Yellow line shows remnant alveolar epithelium. The tumor consists of small lymphoid cells admixed with somewhat larger plasmacytic cells characterised by ample, slightly basophilic cytoplasm and an excentric nucleus.

A 74-year-old man presented to the pulmonologist with a subsolid nodule in the right lower lobe as an incidental finding on abdominal CT. Chest CT was performed six weeks later and showed persistence of the subsolid lesion in the right lower lobe (Figure 1A). The lesion has a predominant ground glass appearance since normal lung architecture with no clear solid areas in mediastinal window (Figure 1B). Because of the large size of the lesion, ^18^F-FDG-PET was performed (Figure 2) and showed moderate FDG uptake. There were no mediastinal or hilar adenopathies and no suspicious extrathoracic findings. Due to persistence of the findings in combination with absence of infection, the patient was referred for surgery. Histopathologic examination showed an extranodal marginal zone lymphoma of mucosa-associated lymphoid tissue (Figure 3).

## Comment

With the widespread use of multidetector CT (MDCT) subsolid nodules are more frequently encountered in daily practice. Subsolid nodules comprise both pure ground-glass and part-solid nodules. The most common diagnosis of persistent subsolid nodules is adenocarcinoma and its precursors [1]: atypical adenomatous hyperplasia, adenocarcinoma in situ, minimally invasive adenocarcinoma and lepidic predominant adenocarcinoma [2]. Other differential diagnoses include focal inflammation, fibrosis, organizing pneumonia and primary pulmonary lymphoma.

Primary pulmonary lymphoma is very rare, accounting only for up to 0.4% of all lymphoma. Most common are non-Hodgkin lymphoma subtypes including extranodal marginal zone lymphoma (EMZL), mucosa-associated lymphoid tissue (MALT) and diffuse large B-cell lymphoma (DLBCL). An association with autoimmune disorders such as Sjögren syndrome, multiple sclerosis, rheumatoid arthritis or systemic lupus erythematosis is known [3]. Clinical findings can hardly differentiate between different etiologies and patients are often asymptomatic [4].

Lymphoma is one of the great mimickers with numerous different imaging features depending on the type of lymphoproliferative disorder. EMZL can present in different ways, both with solitary and multiple lesions. In the majority of cases (>70%) lesions are multiple and bilateral [5]. More common imaging findings include ill-defined solid nodules, cavitating lesions, consolidation, interlobular septal thickening and centrilobular micronodules. Mediastinal adenopathies are usually not associated. The presence of an air bronchogram is very common and bronchial dilatation in an area of consolidation might be a more specific feature for primary pulmonary lymphoma. Presentation as ground glass abnormalities has been described, but in these cases lesions are mostly ill-defined and multiple [4,5,6,7,8]. EMZL or primary lymphoma presenting as solitary subsolid, lesion is rare [1]. The role of PET in staging MALT lymphoma has been unclear and controversial but in contrast to most extrathoracic sites, pulmonary MALT lymphomas are often ^18^F-FDG-avid tumors [8].
